# Longitudinal Grouping of Target Volumes for Volumetric-Modulated Arc Therapy of Multiple Brain Metastases

**DOI:** 10.3389/fonc.2021.578934

**Published:** 2021-06-30

**Authors:** Yingjie Xu, Junjie Miao, Qingfeng Liu, Peng Huang, Pan Ma, Xinyuan Chen, Kuo Men, Jianping Xiao, Jianrong Dai

**Affiliations:** Department of Radiation Oncology, National Cancer Center/National Clinical Research Center for Cancer/Cancer Hospital, Chinese Academy of Medical Sciences and Peking Union Medical College, Beijing, China

**Keywords:** longitudinal-grouping, multiple brain metastases, VMAT, single-isocenter, decrease unnecessary exposure

## Abstract

**Purpose:**

Treatment of multiple brain metastases with single-isocenter volumetric modulated arc therapy causes unnecessary exposure to normal brain tissue. In this study, a longitudinal grouping method was developed to reduce such unnecessary exposure.

**Materials and Methods:**

This method has two main aspects: grouping brain lesions longitudinally according to their longitudinal projection positions in beam’s eye view, and rotating the collimator to 90° to make the multiple leaf collimator leaves conform to the targets longitudinally group by group. For 11 patients with multiple (5–30) brain metastases, two single-isocenter volumetric modulated arc therapy plans were generated using a longitudinal grouping strategy (LGS) and the conventional strategy (CVS). The prescription dose was 52 Gy for 13 fractions. Dose normalization to 100% of the prescription dose in 95% of the planning target volume was adopted. For plan quality comparison, Paddick conformity and the gradient index of the planning target volume, and the mean dose, the V_100%_, V_50%_, V_25%_, and V_10%_ volumes of normal brain tissue were calculated.

**Results:**

There were no significant differences between the LGS and CVS plans in Paddick conformity (p = 0.374) and the gradient index (p = 0.182) of the combined planning target volumes or for V_100%_ (p = 0.266) and V_50%_ (p = 0.155) of the normal brain. However, the V_25%_ and V_10%_ of the normal brain which represented the low-dose region were significantly reduced in the LGS plans (p = 0.004 and p = 0.003, respectively). Consistently, the mean dose of the entire normal brain was 12.04 and 11.17 Gy in the CVS and LGS plans, respectively, a significant reduction in the LGS plans (p = 0.003).

**Conclusions:**

The longitudinal grouping method can decrease unnecessary exposure and reduces the low-dose range in normal brain tissue.

## Introduction

Brain metastases (BMs) are the most common type of intracranial tumors. About 20–40% of patients with cancer develop BMs in their tumor history (1), and multiple BMs are present in approximately 70% of patients with BMs. With recent advances in medical care, chemotherapy, and targeted therapies, overall survival has improved in patients with cancer. With advances in the control of systemic disease, treatment of BMs has become a greater challenge for oncologists (2).

With the continuous development of stereotactic radiosurgery (SRS) and hypofractionated stereotactic radiotherapy (HFSRT) technologies, several studies have found that patients with 5–10 BMs showed similar overall survival to patients with two to four BMs who were treated with SRS (3–5). The SRS and HFSRT has been an effective choice for patients with five or more BMs, especially those who have previously been treated with whole brain radiotherapy.

Traditionally, multiple BMs have been treated individually with SRS or HFSRT. For each lesion, the plan employs one isocenter with several arcs or static beams from a linac or uses several focuses with Gamma Knife shots. When the number of BMs reaches five, the duration of a complete treatment can be many hours. Furthermore, planning is more difficult and requires more care with an increased number of BMs.

Volumetric modulated arc therapy (VMAT), which has been developed in the past decade, has been widely used in tumor treatment at various sites because it produces highly conformal dose distributions and has short treatment delivery times (6). Clark GM et al. (7) contended that single-isocenter VMAT plans can deliver conformity equivalent to that of multiple-isocenter VMAT techniques. In recent years, many studies (8–11) have verified the quality of single-isocenter VMAT plans, and they have been an option in SRS or HFSRT for the treatment of multiple BMs.

However, two problems are introduced by employing single-isocenter VMAT in the treatment of multiple BMs using SRS or HFSRT. One problem is how to manage the rotational uncertainties of the patient setup. Many studies (12–15) have focused on this problem and provided advice to address it. Faught AM et al. (13) suggested that clinical medical physicists revisit the quality assurance tolerances of gantry and multi-leaf collimator (MLC) angles. Miao J et al. (15) proposed the method of expanding the nonuniform gross target volume (GTV) or clinical target volume by adding a planning target volume (PTV) margin. The other problem is how to reduce unnecessary exposure of normal brain tissue. In multiple-target single-isocenter VMAT treatment planning, it is common for a pair of targets to share MLC leaf pairs when they are aligned along the direction of MLC leaf travel [i.e., the “island-blocking” problem proposed by Jun Kang (16)]. The island-blocking problem and the larger jaw openings used in VMAT plans result in increased leakage of the dose between the leaves, which is the main reason for the increase in unnecessary exposure of normal brain tissue. Some researchers (16, 17) have proposed some new algorithms to select the optimal couch and collimator angles to reduce the island-blocking problem. However, those methods require couch rotation during treatment, which would not only increase treatment time but also introduce new errors during the patient setup procedure. If such methods are employed, the problem of increased dose leakage between the leaves caused by the larger jaw openings in single-isocenter VMAT plans still exists. Therefore, the existing methods have limitations.

Helical tomotherapy (HT) employs a small 6-MV linac mounted on a ring gantry. It benefits from its binary MLC, which is perpendicular to the transverse plane of the body and the helical treatment mode. It has excellent dose modulation ability and can deliver a complex dose distribution. The island-blocking problem and the larger jaw opening issue no longer exist when patients with multiple BMs undergo helical tomotherapy.

Inspired by the treatment mode of HT, we developed a longitudinal grouping method to reduce unnecessary exposure of normal brain tissue without couch rotation in single-isocenter VMAT technology for treatment of multiple BMs by SRS or HFSRT. The method can reduce the low-dose range of normal brain tissue, and it is easy to implement.

## Materials and Methods

### Method Description

**Figure 1:**
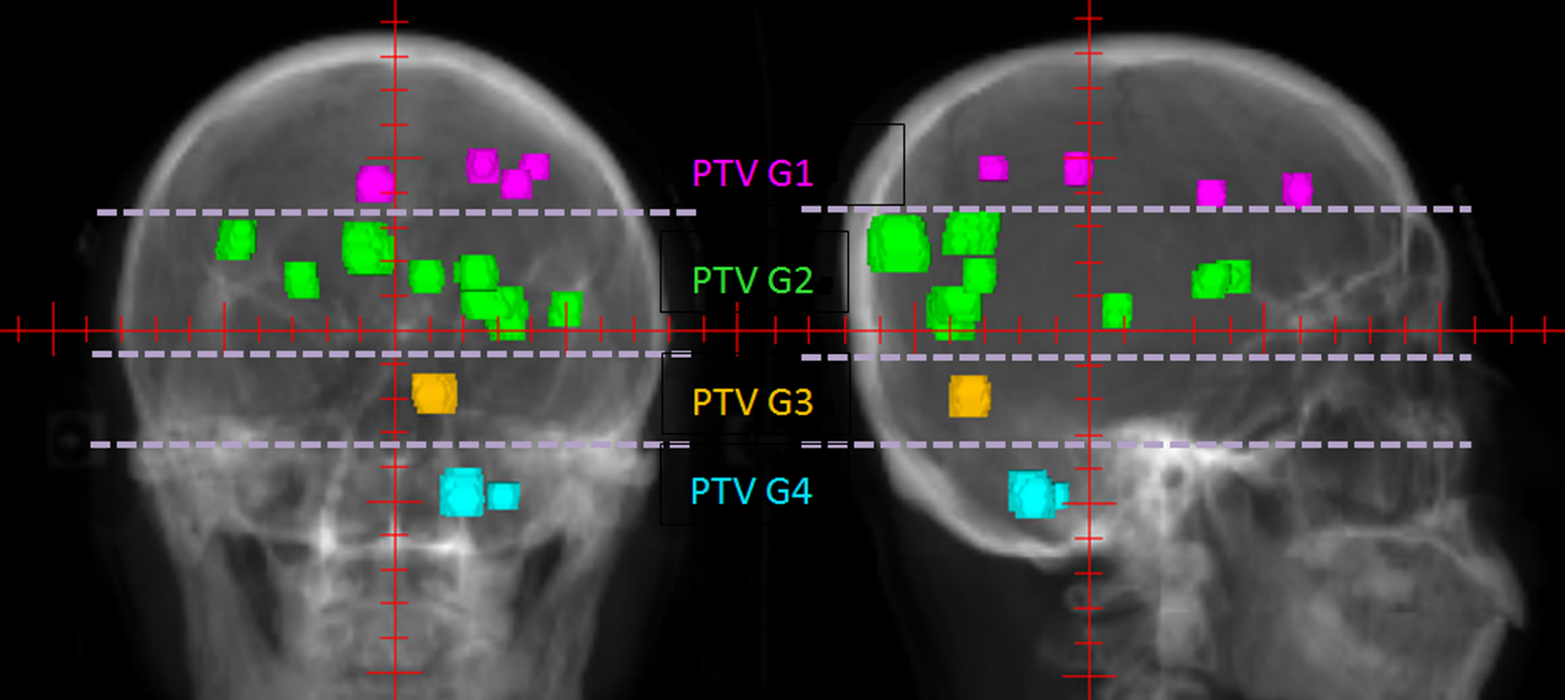
An example of PTV groups in 0° and 90° BEVs. The patient had 14 BMs, which were longitudinally divided into four groups marked by different colors according to their longitudinal projections.

In this study, we developed a longitudinal grouping method to reduce the impact of the island-blocking problem and the larger jaw opening problem in single-isocenter VMAT treatment of multiple BMs. In this method, multiple BMs were divided into several groups according to their locations. Then, treatment arcs were added, and dose optimization was performed. One therapeutic arc was added to each group. The number of therapeutic arcs depended only on the group number, not on the number of BMs in each group. The grouping technique is the most important aspect of this method. In general, the longitudinal positions of the targets on the beam’s eye views (BEVs) throughout all 360° of beam angles are the primary consideration. If the targets’ longitudinal projections overlap when the gantry rotates, they can be grouped together. If a target is located between two groups, and the two groups have targets that overlap with it, group assignment is more difficult. In such cases, the target can be viewed on CT images to observe its relationship to adjacent targets, and then it can be classified into the group that is closest to it on the CT images. After the grouping process, it should be verified that the distance between the adjacent surfaces of the longitudinal projections of any two lesions in the same group does not exceed 1 cm. **Figure 1** is a grouping example shown in BEV. Then, the collimator can be rotated to 90° to align the MLC conformal to the targets in the longitudinal direction group by group, analogously to the treatment mode of HT. The difference is that in this method, the MLC leaf can stay in any position in the field, whereas the MLC leaves have only on and off modes in HT.

**Figure 2:**
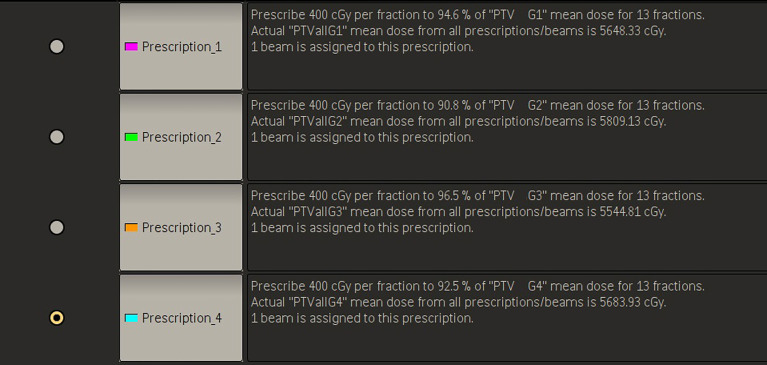
Example prescription settings.

The method has four planning steps. First, all of the multiple BMs are grouped. For each patient, all BMs are composited as a structure named PTVall, and they are divided into several groups longitudinally according to the above grouping method. The associated lesions in each group are combined into a structure named PTV Gnumber, where PTVall is divided into PTV G1, PTV G2, PTV G3, etc. (**Figure 1**). Each PTV group corresponds to a prescription, and the prescriptions are also named according to the corresponding numbers of the PTV groups (e.g., prescription1, prescription2). **Figure 2** shows four prescriptions for an example patient. Second, the isocenter is set, and the first arc is added. The isocenter is positioned at the centroid of all the targets. After the isocenter is set, the first full arc is added and combined with the first prescription in either the clockwise or counter-clockwise direction. The collimator of the arc is set to 90° to ensure that the MLC leaves longitudinally conform to the lesions in the first group. Third, the first arc is optimized. Only the lesions in the first group are added as the target objects. The organs-at-risk (OARs), the rings (i.e., the rings around PTVall), and normal tissue are set as constrained objects. Fourth, another arc is added and optimized group by group. After the optimization of the first lesion group, the second arc is added, the collimator is rotated to 90°, the second group of lesions replaces the first lesion group as the new target objects, and the constrained objects are adjusted according to the new situation. This process is repeated until all lesion groups are added and optimized.

### Patient Selection and Treatment Planning

To evaluate the method’s effectiveness, 11 patients with 5–30 previously treated BMs were retrospectively studied in this work.

According to our clinical practice, patients were treated with HFSRT, and the prescription dose was 52 Gy delivered in 13 fractions for each patient. The CT images were acquired on a Somatom Definition AS 40 (Siemens Healthcare, Forchheim, Germany) or Brilliance CT Big Bore (Philips Healthcare, Best, Netherlands) system with 2-mm slice thickness. The MR images were fused with the CT images for GTV contouring, and the PTVs were derived using a 2-mm expansion from the GTVs. Each patient’s PTVs were combined into a PTVall for plan evaluation. The mean volume of PTVall was 24.49 cm^3^ (2.93–62.51 cm^3^).

In each patient, two VMAT plans were generated for comparison to verify the method’s ability to reduce the low-dose range of exposure to normal brain tissue. One plan was designed using the longitudinal grouping strategy devised in this work (named the LGS plan), and the other plan was developed using the conventional strategy, in which all lesions were optimized simultaneously (named the CVS plan). The isocenter and number of VMAT arcs were identical between the CVS and LGS plans. Except for the fact that PTVall was set as the target of optimization, the other constraint objectives were consistent with the final optimization objectives in the LGS plan, and the structures of the constrains were the same in the two planning methods. All plans were designed using the Pinnacle version 16.2 (Philips Healthcare, Best, Netherlands) treatment planning system and the adaptive convolution algorithm. We used a 6-MV flattening filter-free photon beam with a maximum dose rate of 1,400 MU/min. The MLC with 2.5-mm leaves (Varian HD120) was used for planning and delivery, and each plan was calculated with high-resolution dose grid spacing of 2 mm. All plans were normalized so that 95% of the PTVall volume received 100% of the prescription dose.

### Plan Evaluation and Comparison

The dosimetric parameters of PTVall and the OARs were derived from the dose volume histograms for plan evaluation. According to International Commission on Radiation Units and Measurements reports 83 (18) and 91 (19), the conformity index (CI) and gradient index (GI) were quantitatively assessed as tumor evaluation parameters. The CI represents the degree to which the prescription dose region conforms to the surface of PTVall, and it is calculated using the Paddick formula (20): (TVPV)^2^/(TV × PV), where TVPV represents the volume of PTVall, which is covered by the prescription dose; TV represents the volume of PTVall; and PV represents the prescription isodose volume. The GI is used to evaluate the dose falloff, and it was defined as PV_half_/PV, where PV_half_ denotes the volume enclosed by the isodose surface of half the prescription dose, and PV is the volume enclosed by the prescription isodose surface. The median absorbed dose (D_50%_), the near maximum dose (D_2%_), and the near-minimum dose (D_98%_) of PTVall were recorded for target evaluation.

To evaluate the method’s effectiveness at minimizing unnecessary exposure to normal brain tissue, the dose received by normal brain tissue was also recorded for statistical analysis, including V_100%_, V_50%_, V_25%_, V_10%_, and D_mean_ of normal brain. The decreased proportions of V_25%_ (1 − V_25%, LGS plan_/V_25%, CVS plan_), V_10%_ (1 − V_10%, LGS plan_/V_10%, CVS plan_), and D_mean_ (1 − D_mean, LGS plan_/D_mean, CVS plan_) of normal brain in LGS plans were calculated, and the relationships between the decreased proportions and the number of lesions were analyzed.

### Statistical Analysis

Paired Wilcoxon signed rank two-sided tests were performed on all datasets with IBM SPSS Statistics 19 (SPSS, Inc., Chicago, IL, USA). Individual comparisons between dosimetric parameters were performed, and p-values of <0.05 were considered significant.

## Results

**Figure 3:**
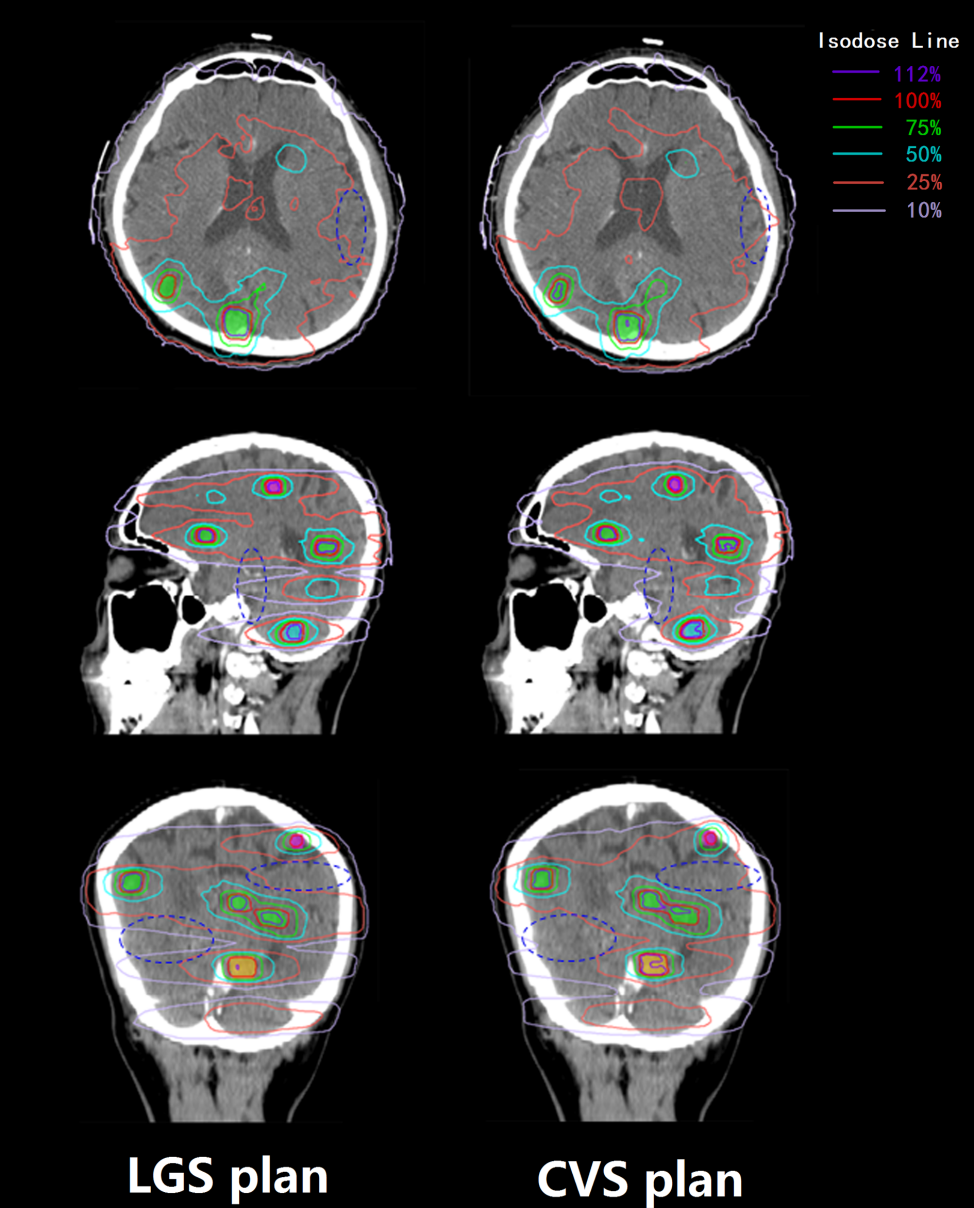
Transverse, sagittal, and coronal dose distributions of two plans for an example patient. The patient had 14 BMs, which were divided into four groups. The 100, 75, and 50% prescription isodose lines did not show much difference between the two plans, but the differences between the 25 and 10% prescription isodose lines between the two plans were obvious. The dark blue ovals mark the areas where there is a significant difference in dose distribution between the two plans.

**Figure 4:**
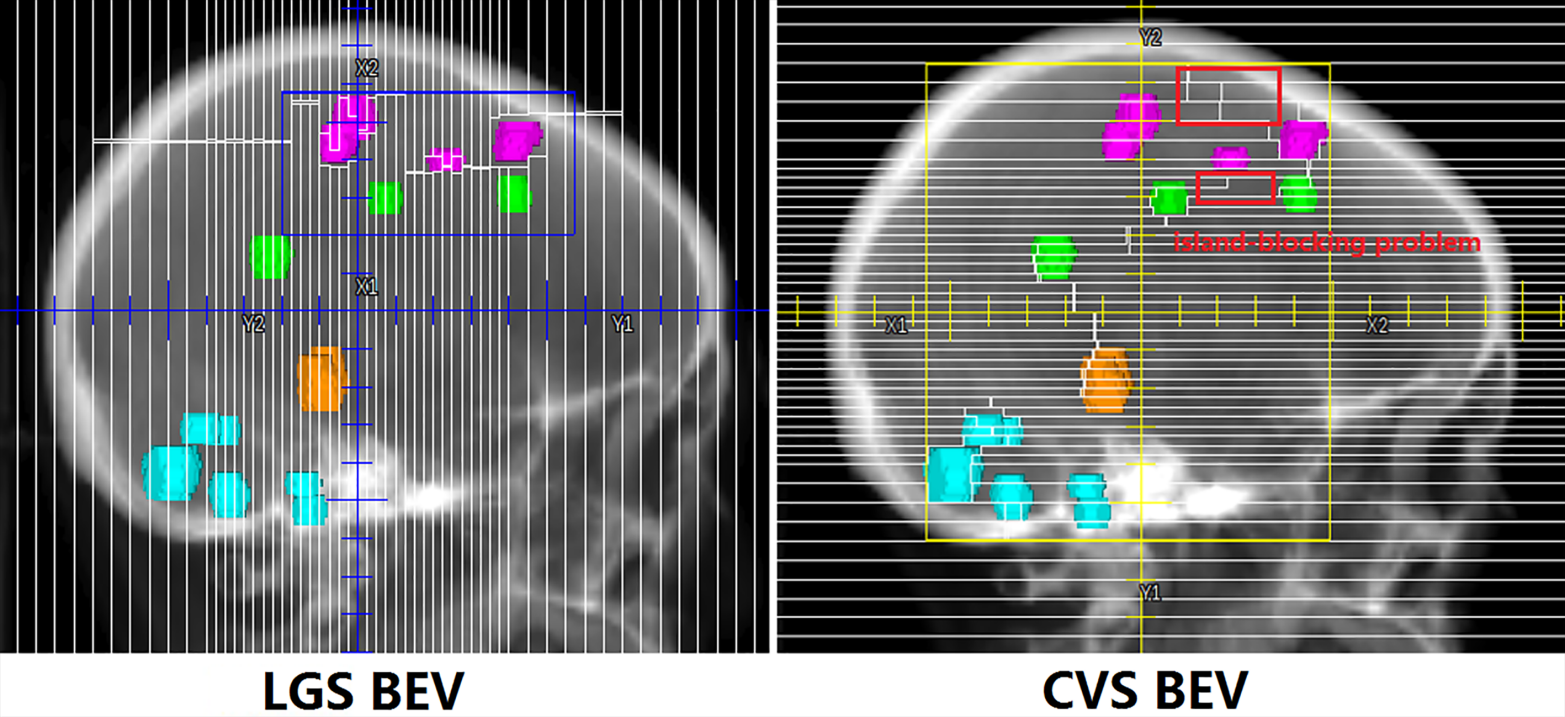
One segment of LGS and CVS in BEV. The control points in the LGS plan are shaped like narrow strips that do not have the island-blocking problem. However, in the CVS plan, the issues of the island-blocking problem and large jaw opening inevitably arise.

All plans achieved 95% coverage of PTVall. **Figure 3** shows representative axial, sagittal, and coronal dose distributions for the two plans. The LGS plan achieved better low-dose distribution. By checking the BEV of each arc (see **Figure 4**), we found that all of the control points of the LGS plan were shaped like narrow strips and did not have the island-blocking problem, whereas the jaw opening was much larger for the control points of the CVS plan, and the island-blocking problem could not be avoided in the CVS plan. **Table 1** contains a summary of the plan evaluation parameters and the respective descriptive statistics.

**Table 1:** Statistics of plan evaluation parameters for the two plan types.

		LGS plan			CVS plan			P
		Range	Median	Mean	Range	Median	Mean	
PTVall	D2%	6,026–6,365	6,229	6,210.55	5,820–6,506	6,253	6,236.82	0.534
	D98%	5,048–5,103	5,072	5,074.36	4,983–5,119	5,035	5,037.45	0.006
	D50%	5,583–5,805	5,689	5,690.91	5,569–5,950	5,755	5,760.82	0.041
	CI	0.69–0.86	0.80	0.80	0.65–0.86	0.79	0.79	0.374
	GI	5.08–8.53	6.04	6.35	4.95–8.80	6.22	6.53	0.182
Normal brain	V100%	0.24–7.81	1.02	1.61	0.20–11.28	1.11	2.13	0.266
	V50%	18.85–367.44	91.95	129.72	18.92–383.79	91.07	139.26	0.155
	V25%	87.81–1,047.50	434.11	511.37	100.48–1,048.71	505.99	543.72	0.004
	V10%	348.24–1,389.17	921.81	928.45	403.40–1,414.54	1,154.37	1,015.68	0.003
	Dmean	398.50–1,829.70	1,079.80	1,116.68	475.50–1,954.50	1,184.90	1,204.35	0.003

**Figure 5:**
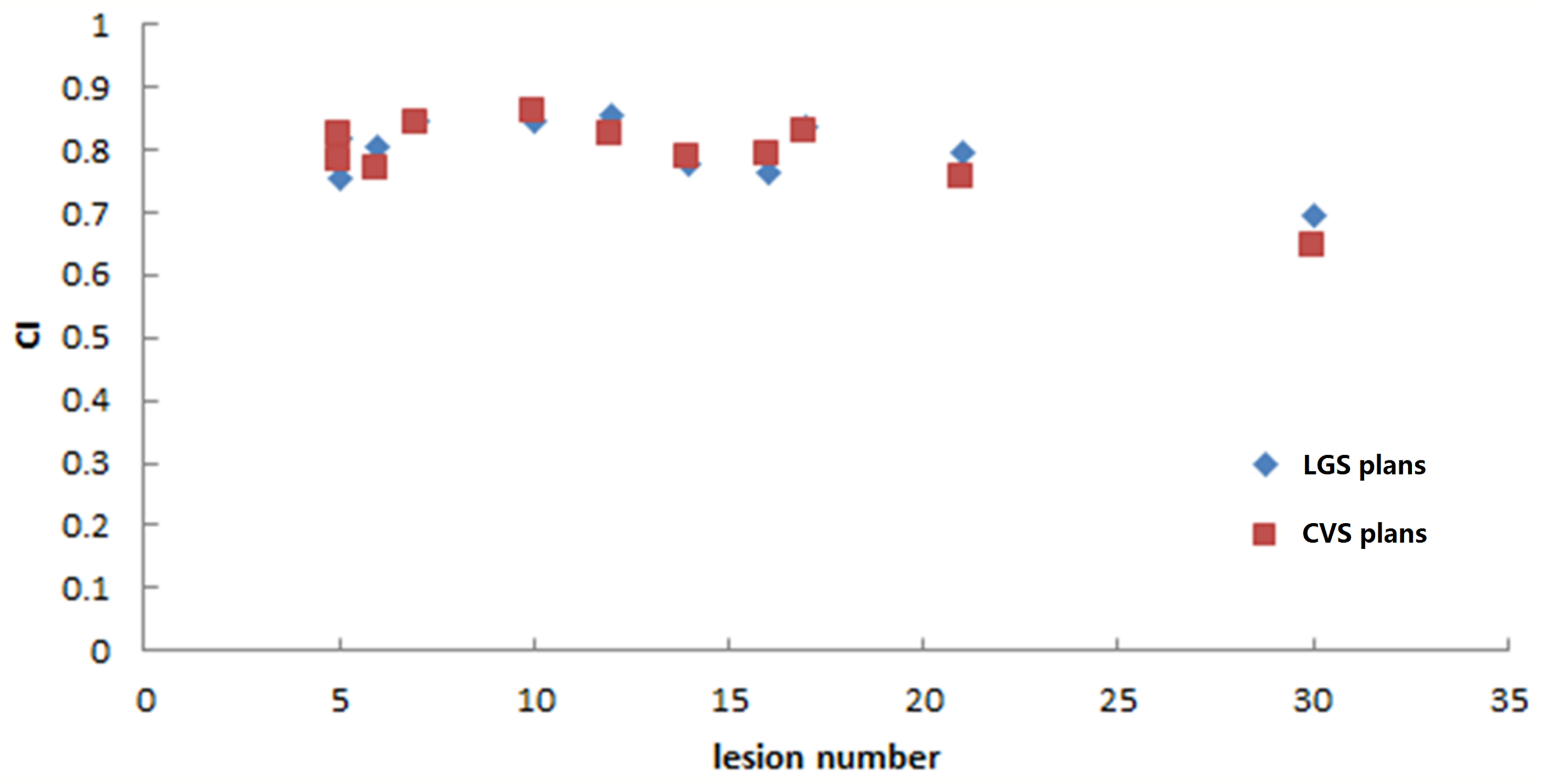
Conformity values of the two plans for the 11 patients.

**Figure 6:**
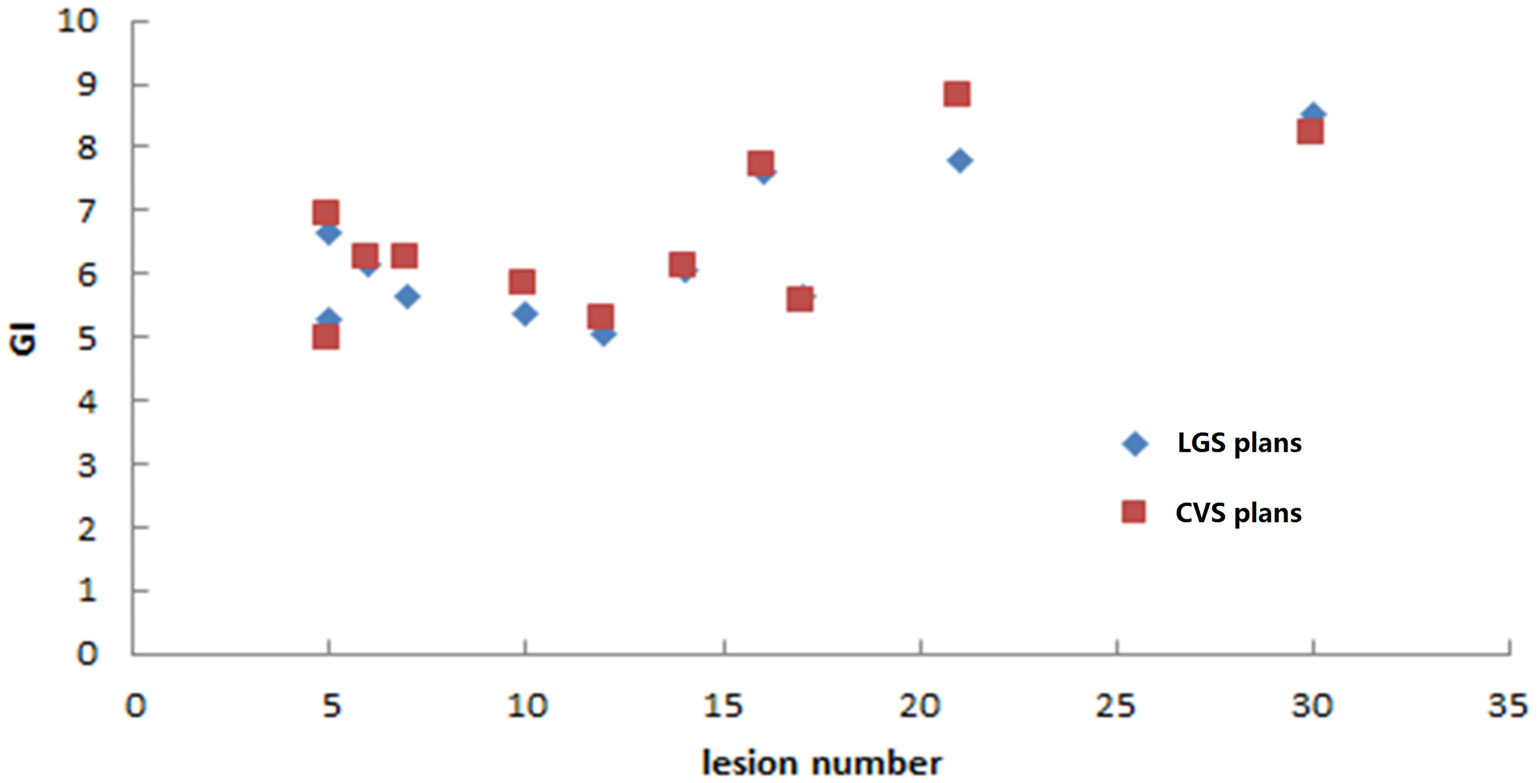
Gradient index values of the two plans for the 11 patients.

The LGS plans achieved a similar level of conformity to that of the CVS plans (CI = 0.80 ± 0.05 and 0.79 ± 0.06, respectively; p = 0.374). There was also no significant difference in GI between the plans (LGS: 6.35 ± 1.15; CVS: 6.53 ± 1.24; p = 0.182). **Figures 5** and **6** show the relationship between CI value, GI value, and lesion number: there was no significant correlation between CI, GI, and the number of BMs. There was also no significant difference in the near-maximum dose (D_2%_) of PTVall between the two plans (p = 0.534). However, the near-minimum dose (D_98%_) of the LGS plan was slightly higher than that of the CVS plan, and the median absorbed dose (D_50%_) of the LGS plan was slightly but significantly lower than that of the CVS plan (p = 0.006 and p = 0.041, respectively).

Consistently with the equivalence in GI values, no significant difference between the two plans resulted for V_100%_ and V_50%_ of normal brain: 1.61 ± 2.17 cm^3^ and 129.72 ± 109.82 cm^3^, respectively, for LGS plans and 2.13 ± 3.26 cm^3^ and 139.26 ± 121.21 cm^3^, respectively, for CVS plans (p = 0.266 and p = 0.155, respectively). Statistically significant improvement in favor of LGS plans was achieved for V_25%_ and V_10%_ of normal brain: the value decreased from (543.72 ± 353.44 cm^3^, 1015.68 ± 366.79 cm^3^), respectively, for CVS plans to (511.37 ± 342.54 cm^3^, 928.45 ± 385.76 cm^3^), respectively, for LGS plans (p = 0.004 and p = 0.003, respectively). Consistently, the LGS plans’ mean dose of normal brain tissue (1116.68 cGy) was significantly lower than that of CVS plans in statistics (1,204.35 cGy; p = 0.003).

**Figure 7:**
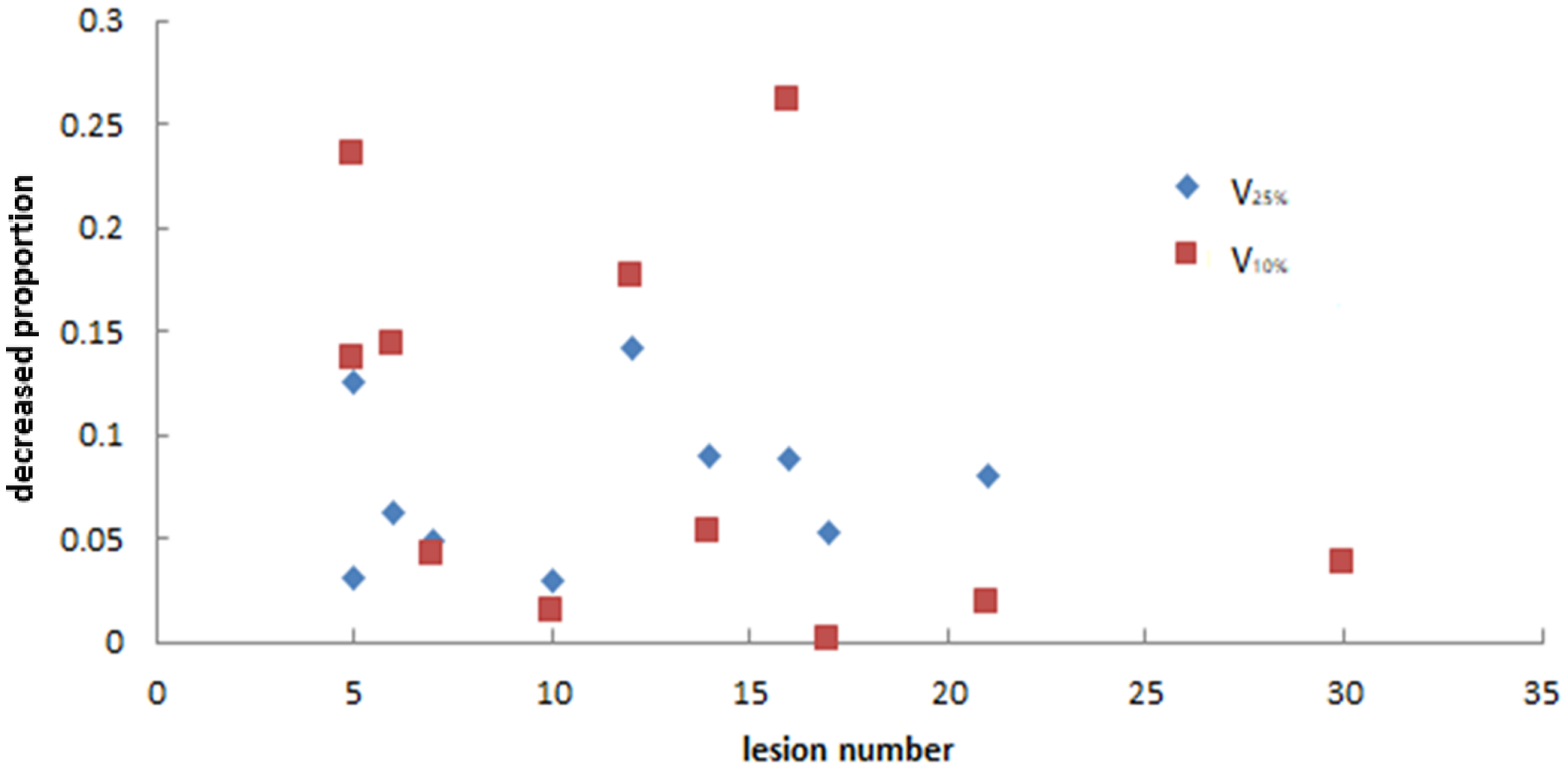
The relationship between the decreased proportion of V_25%_ and V_10%_ of normal brain in LGS plans and the number of lesions. There was no obvious correlation between the two factors.

**Figure 8:**
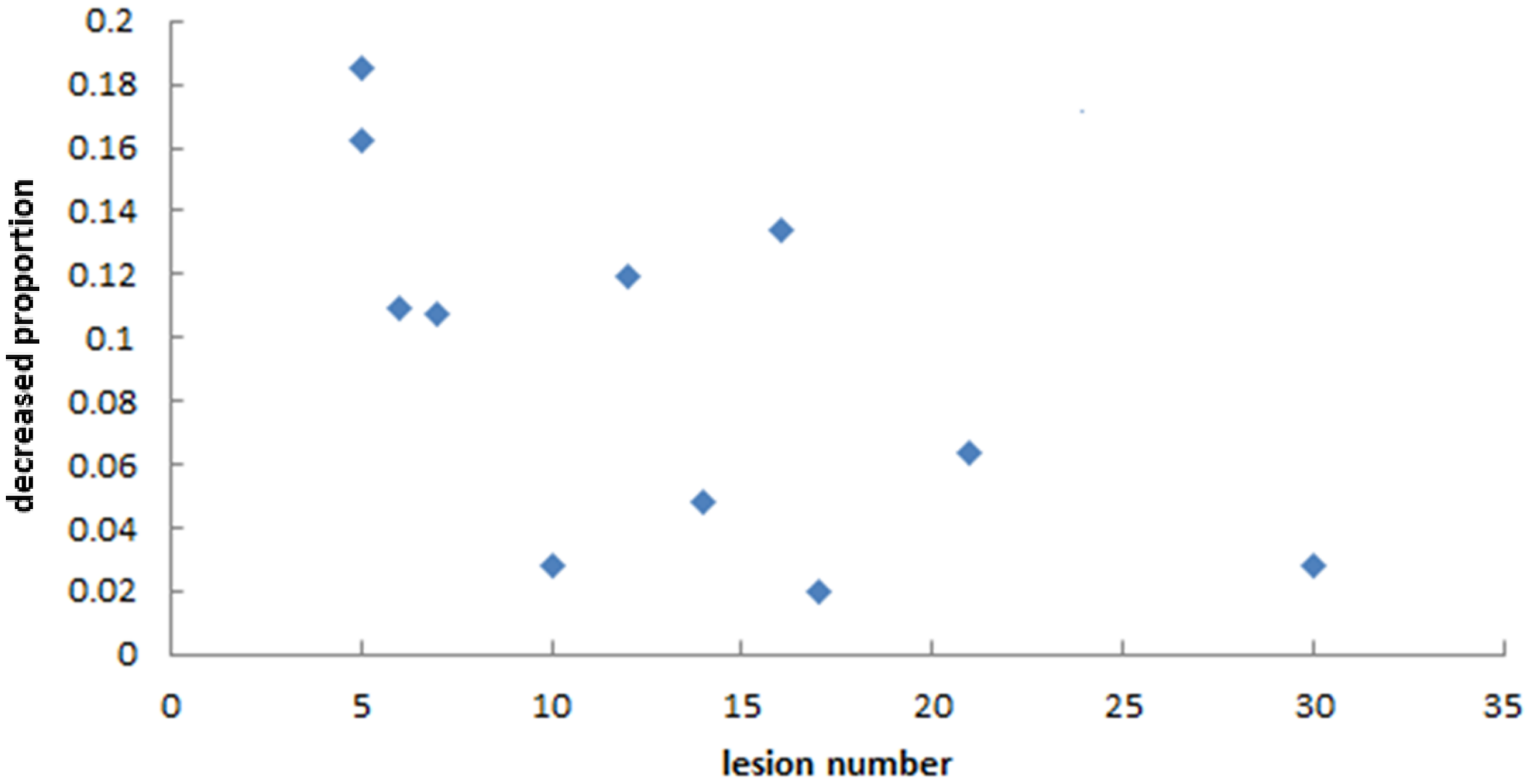
The relationship between the decreased proportion of D_mean_ of normal brain in LGS plans and the number of lesions. It seemed that the decreased proportions in the D_mean_ value of normal brain tended to decrease with an increased number of lesions.

As we mainly focused on evaluating the reduction of unnecessary exposure of normal brain tissue, we determined the relationships between the decreasing proportion of V_25%_, V_10%_, and D_mean_ of normal brain and the number of lesions in the two plans. **Figures 7** and **8** show that there was no particular relationship between the decreased proportions of V_25%_, V_10%_, and D_mean_ of normal brain in the LGS plans and the number of lesions, but the decreased proportions in the D_mean_ value of normal brain tended to decrease with an increased number of lesions.

## Discussion

In this work, we developed a practical method for application in single-isocenter VMAT treatment planning for multiple BMs. This method’s strategy is to mimic the treatment mode of HT in single-isocenter VMAT plans. In our department, HT has been used in multiple BMs radiotherapy and has obtained good clinical results (21). Accordingly, if the collimator setting of the C-arm linac is maintained at 90°, the motion of the MLC leaves would be similar to the motion of binary MLC in HT, but more flexible when the optimized target objective is limited to a group of lesions. Since the maximum adjacent surface distance between the longitudinal projections of lesions in the same group did not exceed 1 cm, even when two or more lesions shared the same MLC leaf pairs, there was no unnecessary exposure to normal brain because there was only a narrow exposure gap between the lesions. Furthermore, each VMAT arc only covered lesions in the same group, which indirectly mitigates the deleterious effects of large jaw-defined field sizes. This reduces the island-blocking problem, and reducing the area of the jaw opening also decreases the leakage dose between the leaves, is which caused by the large jaw opening. The present study’s results verify this reasoning.

The results of comparisons between the two plan types showed no differences in conformity or gradient between the two methods. This may be because except for the first VMAT arc, the other VMAT arcs were optimized with the previously optimized dose, which may affect the optimization results of the current arc. Although there was no statistical difference between the two plan types’ average values of CI and GI, the conformity and gradient of the LGS plan were slightly better than those of the CVS plan.

The method proposed in this study decreased the low-dose region of normal brain tissue in some patients, but in some patients, the magnitude of the decrease was small. We can analyze this method’s effectiveness according to the number and location distribution of the BMs. First, **Figure 8** shows that this method’s effectiveness may decrease with an increased number of BMs, in line with our expectations. When the number of BMs increased, most BMs were adjacent to each other on BEVs. Even if the VMAT plan is created by conventional methods, this reduces the island-blocking problem, and most of the leakage between MLC leaves is also irradiated into tumors, reducing the impact on the low-dose range of normal brain tissue. The locations of BMs are also an influencing factor. The distribution of targets in BEVs can fall into the following three categories: (a) very concentrated; (b) very scattered; (c) partly concentrated, partly scattered. If the targets are very concentrated (i.e., all BMs show closely adjacent status on BEVs), there is no need to use this method because the impact of the problems mentioned above is very small. Thus, the longitudinal grouping method is mainly useful for the other two categories. Furthermore, if there is a large distance between the BMs in the cephalo-caudal direction, a large area of normal brain tissues between those targets may be exposed to low-dose irradiation if the VMAT plan is devised by conventional methods. In addition, the low dose region may be larger if the jaw is fixed during optimization. The longitudinal grouping method would be more advantageous in the latter situation.

Although the proposed method was applied to multiple BMs treated with VMAT, it can also be applied to other situations in which multiple target volumes are treated by either VMAT or IMRT.

In the current method, tumors are grouped manually. This makes the planning process more complicated and time-consuming than the conventional method, in which all of the lesions are planned at the same time. Automatic grouping could solve this limitation, but such methods would require cooperation from the vendors of treatment planning systems. This method can also be applied to non-coplanar situations, but the effects of that specific application require further research.

In conclusion, the longitudinal grouping method can decrease unnecessary exposure and reduces the low-dose range of exposure to normal brain tissue.

## Data Availability Statement

The original contributions presented in the study are included in the article/Supplementary Material; further inquiries can be directed to the corresponding author.

## Ethics Statement

The studies involving human participants were reviewed and approved by Ethics Committee of National Cancer Center/Cancer Hospital, Chinese Academy of Medical Sciences and Peking Union Medical College. Written informed consent for participation was not required for this study in accordance with the national legislation and the institutional requirements. Written informed consent was obtained from the individual(s) for the publication of any potentially identifiable images or data included in this article.

## Author Contributions

Conception and design: YX and JD. Financial support: JD. Provision of study materials or patients: QL and JX. Collection and assembly of data: YX, JM, PH, PM, XC, and KM. Data analysis and interpretation: YX and JM. Manuscript writing: All authors. All authors contributed to the article and approved the submitted version.

## Funding

This work was supported by the National Natural Science Foundation of China (grant number 11875320).

## Conflict of Interest

The authors declare that the research was conducted in the absence of any commercial or financial relationships that could be construed as a potential conflict of interest.
